# Resetting histone modifications during human prenatal germline development

**DOI:** 10.1038/s41421-023-00519-1

**Published:** 2023-02-03

**Authors:** Rui Gao, Shiyang Zeng, Dongxu Yang, Xiaocui Li, Wenqiang Liu, Yawei Gao, Dandan Bai, Linfeng Zhang, Chuan Chen, Yunzhe Kang, Beiying Wang, Wei Hong, Mingzhu Wang, Jiqing Yin, Hong Wang, Qiaolin Deng, Shaorong Gao, Yong Zhang, Jiayu Chen

**Affiliations:** 1 Clinical and Translation Research Center of Shanghai First Maternity & Infant Hospital, Shanghai Key Laboratory of Signaling and Disease Research, Frontier Science Center for Stem Cell Research, School of Life Sciences and Technology, Tongji University, Shanghai, China; 2 Institute for Regenerative Medicine, Shanghai East Hospital, Shanghai Key Laboratory of Signaling and Disease Research, Frontier Science Center for Stem Cell Research, School of Life Sciences and Technology, Tongji University, Shanghai, China; 3 Department of Physiology and pharmacology, Biomedicum B5, Karolinska Institutet, Center for Molecular Medicine, Karolinska University Hospital, Stockholm, Sweden

**Keywords:** Embryonic germ cells, Embryonic germ cells

## Abstract

Histone modifications play critical roles in regulating gene expression and present dynamic changes during early embryo development. However, how they are reprogrammed during human prenatal germline development has not yet been elucidated. Here, we map the genome-wide profiles of three key histone modifications in human primordial germ cells (hPGCs) from weeks 8 to 23 of gestation for the first time by performing ULI-NChIP-seq. Notably, H3K4me3 exhibits a canonical promoter-enriched pattern, though with relatively lower enrichment, and is positively correlated with gene expression in globally hypomethylated hPGCs. In addition, H3K27me3 presents very low enrichment but plays an important role in not only dynamically governing specific bivalent promoters but also impeding complete X chromosome reactivation in female hPGCs. Given the activation effects of both global DNA demethylation and H3K4me3 signals, repressive H3K9me3 and H3K27me3 marks are jointly responsible for the paradoxical regulation of demethylation-resistant regions in hPGCs. Collectively, our results provide a unique roadmap of three core histone modifications during hPGC development, which helps to elucidate the architecture of germ cell reprogramming in an extremely hypomethylated DNA environment.

## Introduction

Mammalian primordial germ cells (PGCs) are embryonic precursors that can give rise to highly specialized, differentiated gametes. The proper development of germ cells is considered essential for faithfully carrying both genetic and epigenetic information from one generation to the next, which maintains the hereditary continuity of a species^1^. PGCs experience a series of specialized cellular processes, including migration, localization to genital ridges, sexual differentiation, meiosis, and mature gamete formation^2,3^ throughout their development. Both the transcriptome and epigenome are extensively reset in parallel within these critical processes^4^, which supports the subsequent establishment of totipotency after fertilization. However, due to limited number of these cells, little is known about the exact changes that occur during human PGC (hPGC) development.

In the past several years, with the improvement of micro-omics technologies and related bioinformatics analyses, extensive and accurate chromatin remodeling information, including data on the transcriptome, DNA methylome and chromatin accessibility of fetal hPGCs, has been obtained by our group and others^5–11^. The transcriptomes of the individual hPGCs of both sexes show stepwise gene expression changes and exhibit an asynchronous and heterogeneous nature within their highly orchestrated migration, mitosis, meiosis and gametogenesis^9^. Meanwhile, the chromatin accessibility and DNA methylation state in hPGCs are generally similar to those in mice at comparable developmental stages, suggesting the evolutionary conservation of these reprogramming dynamics in these two species^7^. However, current studies on histone modifications in hPGCs are largely based on immunofluorescent staining, their distribution, dynamics and correlation with other epigenetic modifications such as DNA methylation and chromatin accessibility during development are largely unclear. In addition, whether they are involved in the regulation of transcription and certain specific events (for example, X chromosome regulation and DNA demethylation escapees) in the hypomethylated hPGCs remains to be answered.

It was reported that human preimplantation embryos possessed a unique X-chromosome dosage compensation mechanism^12,13^. For hPGCs, it was unexpected to find that XIST noncoding RNA is expressed throughout human germline development, which is not restricted to female hPGCs^5^. Additionally, the inactivated X chromosome is reactivated in female hPGCs in Wk4 (week 4) and thereafter^6,10^, which indicates that XIST might not be responsible for X chromosome regulation during this particular developmental process. A more interesting finding was that the total expression level of the genes on the X chromosomes of female hPGCs is increased by 1.6-fold, rather than 2-fold, over that in their male counterparts^6^, which further indicates the occurrence of X-chromosome dampening (XCD) in hPGCs. Collectively, these studies highly suggested that certain epigenetic mechanisms must be in place to either increase gene expression from the single X of males or repress gene expression from the double X chromosomes of females. Since the genome exhibits extensive DNA demethylation, repressive histone modifications such as H3K27me3 and H3K9me3 might largely contribute to X-chromosome regulation during hPGC development.

Genome-wide DNA demethylation is another significant phenomenon that takes place during prenatal hPGC development. It was demonstrated that global DNA demethylation occurs before hPGCs colonize the gonads and that DNA methylation reaches the basal level in Wk10–11, around the time of sex determination^5,6,10^. However, the global absence of DNA methylation does not lead to excessive transcriptional chaos. Moreover, there is almost no correlation between gene expression and DNA methylation during the corresponding time period^5,10^, indicating that specific chromatin modifications might take the responsibility. Meanwhile, the loss of DNA methylation in hPGCs may trigger the reorganization of repressive chromatin modifications to repress global gene or retrotransposon activation and maintain genome stability. More interestingly, although most genomic regions are hypomethylated, a small proportion of genomic regions have been found to evade full demethylation during hPGC development, which are therefore referred to as DNA demethylation escapees^10^. These regions can be further divided into repeat-poor and repeat-rich escapees. However, despite being partially methylated in a hypomethylated environment, the repeat-rich escapees are unexpectedly activated or show no prominent repression. Whether active or repressive histone modifications participate in this particular event triggers great interests. Thus, the mechanisms underlying demethylation resistance in the human germline need to be carefully interpreted.

Recently, several groups have profiled the histone modifications in mouse PGCs at certain developmental time points during sexual differentiation and meiotic initiation by using the traditional chromatin immunoprecipitation coupled with high-throughput sequencing (ChIP-seq) strategy^14–16^. Besides, we and others have revealed temporally accessible chromatin configurations during mouse and human PGC development^7,8^. Mouse PGCs exhibit high levels of H3K27me3 in genes and retrotransposons that are enriched for developmental and differentiation functions, indicating extensive silencing of key developmental pathways in such germ cells^15^. In addition, the paradoxical coexistence of bivalent H3K4me3 and H3K27me3 domains has been identified and is suggested to play an important role in maintaining developmental genes in a silenced state poised for activation upon subsequent differentiation^14,16^. However, due to the lack of highly sensitive technologies for analyzing precious, low-abundance materials, there is still limited information about the germline-specific properties of histone modifications in hPGCs. It has been indicated that human and mouse PGCs show distinct H3K27me3 dynamics^10,17^. In addition, although DNA demethylation is accompanied by a global depletion of H3K9me2, H3K9me3 probably serves as the main factor in repressing constitutive heterochromatin in hPGCs^6,10^. Since immunofluorescence-based analysis lacks gene-level resolution and may lead to conflicting results, a genome-wide analysis of the chromatin regulatory framework involved in human germ cell specification and differentiation is urgently needed.

Here, by applying ultralow-input micrococcal nuclease-based native chromatin immunoprecipitation followed by sequencing (ULI-NChIP-seq) analysis, we provide high-resolution, genome-wide, comprehensive chromatin data for both male and female fetal hPGCs as well as gonadal somatic cells for the first time during 16 weeks of development, from Wk8 to Wk23 of gestation. We demonstrate that H3K4me3 exhibits a canonical promoter-enriched pattern, and shows a high correlation with transcription. H3K27me3 presents very little enrichment but plays an important role not only in dynamically governing specific bivalent promoters during germline cell development but also in impeding complete X chromosome reactivation in female hPGCs. Moreover, under the synergistic activation effect mediated by global DNA hypomethylation and H3K4me3 signals, repressive histone modifications are jointly responsible for the paradoxical regulation of demethylation-resistant repeat regions in hPGCs. Together, our study reveals that histone modifications specifically function in the regulation of hPGC development under a consistent DNA hypomethylated environment.

## Results

### Genome-wide profiling of histone modification in hPGCs

**Fig. 1:**
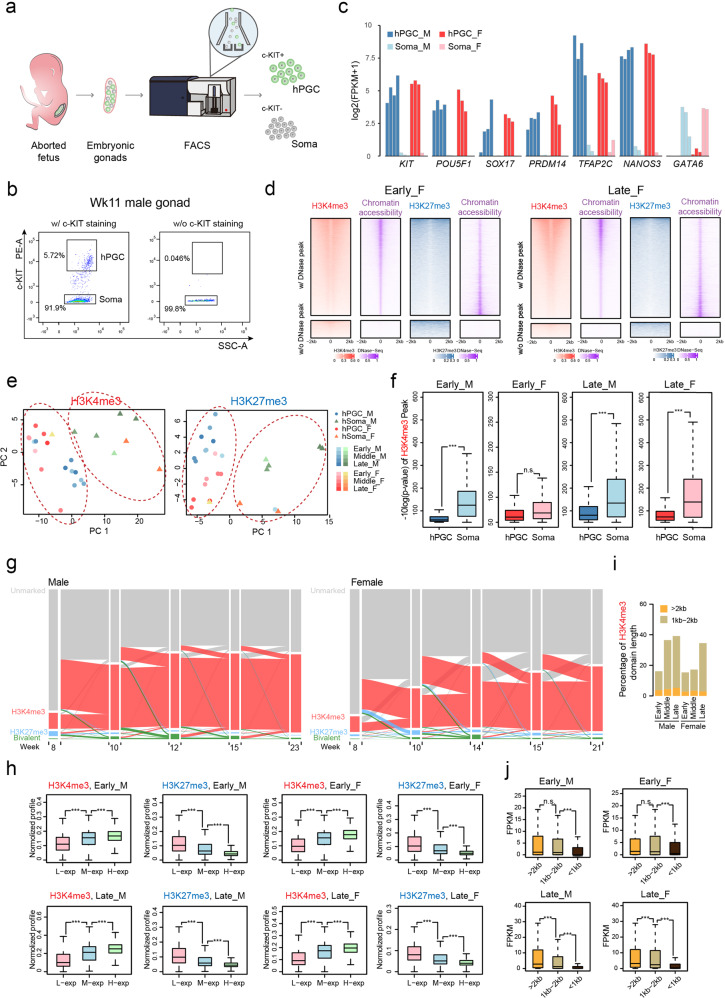
Dynamics of H3K4me3 and H3K27me3 chromatin domains in hPGCs. **a** Schematic of isolating hPGCs (c-KIT–positive) and gonadal somatic cells (soma) (c-KIT–negative) used in ULI-NChIP-seq analysis. **b** Isolation of hPGCs (c-KIT–positive) and somatic cells (Soma, cKIT–negative) from a 11-week (Wk11) male embryonic gonads by FACS with cell-surface marker c-KIT (left). In each experiment, sample with a same treatment but without c-KIT staining (w/o c-KIT) was served as the negative control (right). **c** Barplot showing expression levels of selected marker genes in male hPGC (blue), female hPGC (red) samples and somatic cell samples of both sexes (male, light blue; female, pink). Expression levels are represented as log_2_(FPKM + 1). **d** Heatmaps showing the relationship between histone modification H3K4me3, H3K27me3 and chromatin accessibility on potential cis-elements defined by promoters and DNase-seq peaks in early and late stage female hPGCs. **e** PCA of ChIP-seq profiles on human genome in samples including male (blue) and female (red) hPGCs, male (green) and female (orange) gonadal somatic cells from early (light color) stage to late (dark color) stage. The “Circle” indicates hPGC samples, and the “Triangle” indicates somatic cells. The “Light”-to-“Dark” color indicates biological samples isolated from early-to-late fetal germ cell developmental stages. **f** Boxplots showing comparison of H3K4me3 peak significance level between hPGCs and somatic cells at early and late stage in male and female. **g** Alluvial plot showing the global dynamics of (red) H3K4me3-, (blue) H3K27me3-, (green) bivalent-covered and (gray) unmarked promoters on human genome in hPGC samples of both sexes at indicated time points. **h** Boxplots showing the comparison of H3K4me3 and H3K27me3 profiles on low expression (L-exp), intermediate expression (M-exp) and high expression (H-exp) gene promoters in hPGC samples of both sexes at indicated stage. Profiles are calculated as ChIP-seq RPM. Genes were split equally into low, medium and high expression groups by ranks of expression in each period. **i** Barplot showing the proportion of 1 kb–2 kb (light brown) and > 2 kb (orange) H3K4me3 domains in hPGC samples of both sexes at indicated stage. **j** Boxplots showing the comparison of expression levels between genes, whose promoters are covered by different length of H3K4me3 domains in hPGC samples of both sexes at indicated stage. Expression levels were represented as FPKM. For male, Wk8–10, Wk15 and Wk23 hPGC samples are represented as early, middle and late stage, respectively; for female, Wk8–10, Wk14 and Wk21 hPGC sample are represented as early, middle and late stage, respectively. Soma, gonadal somatic cells, F female, M male, w/ with, w/o without. Student’s *t* test is performed to examine the significant statistical difference between two groups of data in boxplots in (**f**, **h** and **j**). *P < 0.05, **P < 0.01, ***P < 0.001, n.s. not significant.

To investigate genome-wide chromatin dynamics during human prenatal germline development, we isolated gonadal PGCs from genital ridges between Wk8 and Wk23 of age with ethical approval. According to previous studies^5–10^, hPGCs can be well purified by fluorescence-activated cell sorting (FACS) according to higher expression of the surface marker c-KIT (Fig. 1a, b). Meanwhile, c-KIT-negative somatic cells could be also collected to elucidate the PGC-niche interaction during this developmental period. Then, both c-KIT-positive and c-KIT-negative cells were subjected to comprehensive analysis via ULI-NChIP-seq, and we generated the genome-wide profiles of H3K4me3, H3K27me3 and H3K9me3 modifications in each corresponding gestation stage (Supplementary Fig. S1a). These stages encompass the mitotic period of hPGCs after the colonization of the genital ridges, meiotic entry in oogonia and mitotic quiescence in gonocytes^9^. In addition, the transcriptional networks of these cells were identified by RNA sequencing, and the purity of hPGCs and gonadal somatic samples was confirmed with an expanded panel of selected germ cell-expressed genes including *POU5F1*, *SOX17*, *PRDM14*, *TFAP2C* and somatic cell-expressed gene *GATA6* (Fig. 1c and Supplementary Fig. S1b).

In general, the developing male and female genital ridges exhibited discernible morphological differences from Wk10–12 onward (Supplementary Fig. S1c) and showed the loss of nuclear OCT4 deposition (Supplementary Fig. S1d), which agrees with their sexual differentiation^10^. Immunofluorescence analysis clearly indicated that the male genital ridges formed typical testicular structures throughout the gonad as early as Wk10 (Supplementary Fig. S1d). These male germ cells enter mitotic quiescence synchronously^9^ and start to highly express a number of genes related to both spermatogenesis and sexual reproduction (Supplementary Fig. S1e). By contrast, female hPGCs enter meiosis asynchronously for a period of time^9^, and individual follicle-like structures cannot be clearly observed until Wk15 (Supplementary Fig. S1d).

To analyze the chromatin state during hPGC development, we performed peak calling analysis through model-based analysis for ChIP-seq (MACS). We first examined the distribution of the H3K4me3- and H3K27me3-enriched regions and found that both modifications were preferentially enriched in promoter regions, which is consistent with their close association with transcriptional activation and repression, respectively (Supplementary Fig. S1f). Meanwhile, H3K4me3 was positively correlated with the accessible chromatin, whereas H3K27me3 showed a clear negative correlation during the hPGC development (Fig. 1d). Principal component analysis (PCA) indicated that both male and female hPGCs (c-KIT positive) mainly separately clustered from the corresponding somatic cells (c-KIT negative) (Fig. 1e). Moreover, sex differences could be well distinguished throughout human prenatal germline development (Fig. 1e). In contrast, the transcriptome of hPGCs and their corresponding somatic cells in different developmental stages exhibited a more obscure distribution, and mitotic hPGCs before Wk10–11 showed no obvious differences between male and female germ cells^5,6^. Thus, despite the bewildering transcription and consistent DNA hypomethylation observed, histone modifications seem to be prepared for the orchestration of subsequent gametogenesis.

Since extreme genome-wide hypomethylation does not lead to excessive transcriptional excitability, certain activation-related mechanisms may be absent and/or repressive mechanisms must be in place. Here, we showed for the first time that H3K4me3 is less enriched during human germ cell development compared to surrounding somatic cells in both sexes (Fig. 1f). Surprisingly, the global number of H3K4me3-enriched promoters increased rapidly in male hPGCs from Wk10 onward; in contrast, H3K4me3 marks appeared to be established at relatively low levels in female hPGCs (Fig. 1g). The immunofluorescent staining further confirmed the gain of H3K4me3 marks during development (Supplementary Fig. S1g). We further conducted a parallel analysis of H3K4me3 modification among hPGCs, hPGC-like cell (hPGCLC) and human embryonic stem cells (hESCs). Intriguingly, hPGCLCs showed a much similar distribution of H3K4me3 signals with hESCs instead of hPGCs due to their enrichment in key markers and central regulators for embryogenesis but not gametogenesis (Supplementary Fig. S1h).

Genes that were highly expressed in the hPGCs of both sexes during development generally exhibited higher H3K4me3 enrichment. In addition, these highly expressed genes presented not only higher H3K4me3 occupancy but also fewer or no H3K27me3 signals (Fig. 1h). Interestingly, certain non-canonical flat H3K4me3-enriched regions (~2 kb) with relatively low enrichment could be observed at a very early stage, while unique flat H3K4me3-enriched regions with relatively greater enrichment were further established after Wk10 (Fig. 1i and Supplementary Fig. S1i). We then compared the transcriptome activity and width of the H3K4me3-enriched regions in the promoters of individual genes and found that the broader H3K4me3-enriched regions mainly indicated a higher level of gene expression especially in the late development stage (Fig. 1j), similar to what is observed during mouse early embryo development^18^.

### Dynamics of H3K27me3-dependent bivalent chromatin domains in hPGCs

**Fig. 2:**
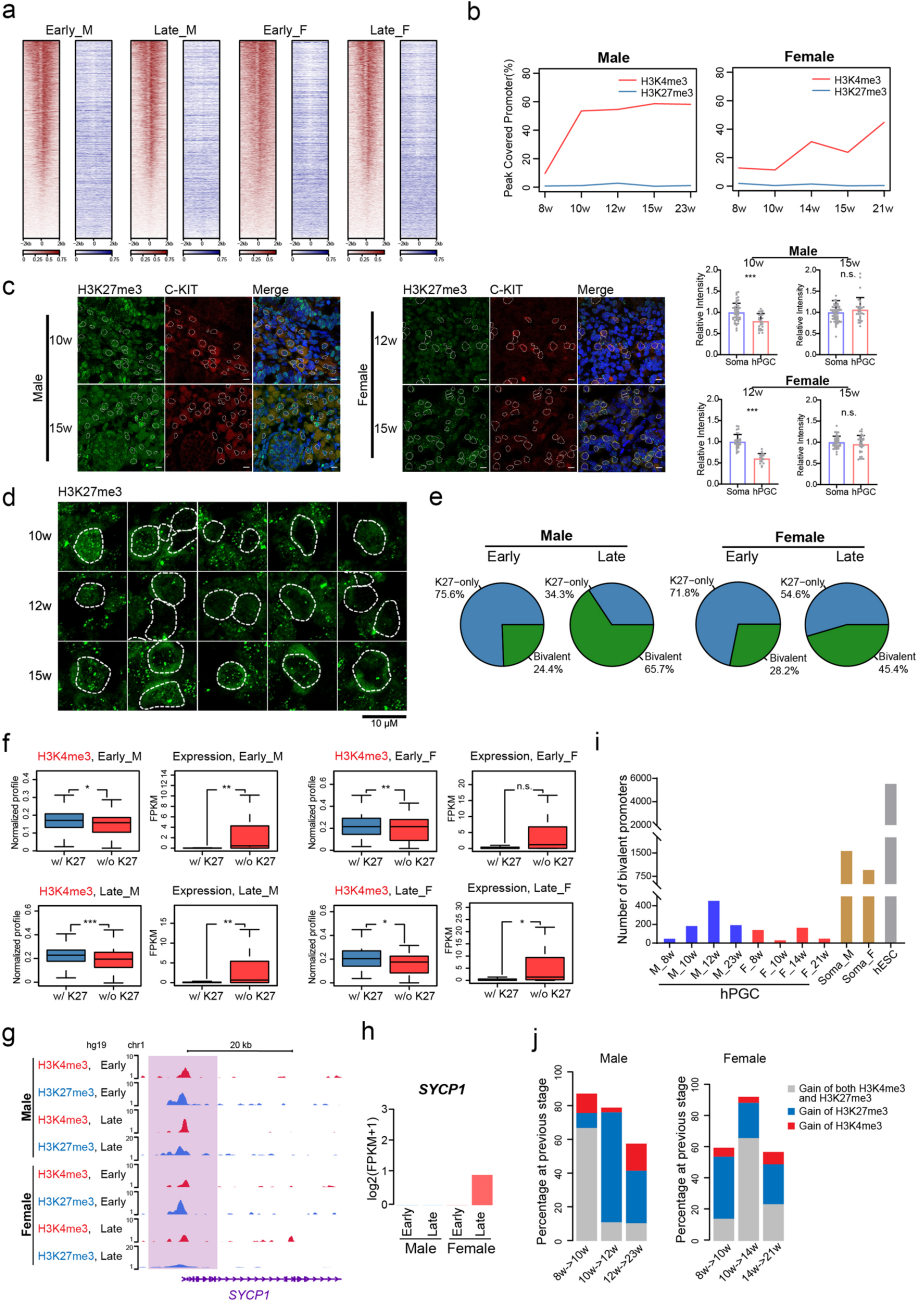
Bivalent chromatin domains in hPGCs. **a** Heatmaps showing the distribution of H3K4me3 (red) and H3K27me3 (blue) profiles around TSS (± 2 kb) in male and female hPGC samples at early and late stage. Signals are sorted by H3K4me3 profiles at each stage and colors indicated ChIP-seq RPM. **b** Line plots showing the global dynamics of H3K4me3 and H3K27me3 covered promoters in male and female hPGC samples at indicated time points. **c** Left, immunofluorescence analysis for H3K27me3 in male and female genital ridge cryosections at indicated time points. The nuclei of c-KIT positive hPGCs are indicated by white dotted line. Scale bars, 10 μm. Right, analysis of relative intensity of H3K27me3 between indicated samples. Each dot represents the relative fluorescence intensity of a single nucleus. Data are represented as the means ± SEM (*n* ≥ 18 single nuclei). **d** Magnified immunofluorescence images showing female hPGCs enriched with strong punctuated H3K27me3 signals. The nuclei of individual c-KIT positive hPGCs are marked by white dotted line. **e** Pie plots showing the proportion of bivalent (green) and H3K27me3-only (blue) promoters within all the H3K27me3 covered promoters in hPGC samples of both sexes at early and late stage. **f** Boxplots showing the H3K4me3 profiles and gene expression levels on promoters with (w/) and without (w/o) H3K27me3 enrichment at early and late stage of male and female hPGCs. H3K4me3 signals at H3K27me3 covered promoters were much higher than those at non-H3K27me3 covered promoters. And genes covered by H3K27me3 signals showed a much lower expression level. Expression levels are represented as FPKM and profiles are calculated as ChIP-seq RPM. **g** Genome browser view of H3K4me3 and H3K27me3 profiles (shaded in light purple) on representative bivalent gene *SYCP1* of both sexes at early and late stage. Profiles are calculated as ChIP-seq RPM and smoothed as 3 pixels window using WashU Epigenome Browser. **h** Barplot showing *SYCP1* expression in hPGCs of both sexes at early and late stage. **i** Barplot showing the number of bivalent promoters in hPGCs of both sexes at indicated time points, gonadal somatic cells and hESCs. Data of hESCs is cited from GSE29611. **j** Sources of gained bivalent genes from previous stage to present stage in male and female hPGC samples. For male, Wk8–10 and Wk23 hPGC samples are represented as early and late stage, respectively; for female, Wk8–10 and Wk21 hPGC samples are represented as early and late stage, respectively. Soma, gonadal somatic cells, F female, M male. Student’s *t* test is performed to examine the significant statistical difference between two groups of data in (**c** and **f**). *P < 0.05, **P < 0.01, ***P < 0.001, n.s. not significant.

Previous studies have indicated that hPGCs are nearly depleted of H3K27me3^6,10^. Here, we further demonstrated the global number of H3K27me3-enriched regions in hPGCs exhibited a much lesser extent in both sexes during all the investigated developmental stages compared to the relatively moderate H3K4me3 signals in the same germ cells (Fig. 2a, b and Supplementary Fig. S2a). In addition, the number of H3K27me3-enriched regions slightly fluctuated during these stages, which differs considerably from the abundant enrichment of H3K27me3 in mouse PGCs (Supplementary Fig. S2b)^19^. The immunofluorescent staining analysis further demonstrated that hPGCs of both sexes showed a lower H3K27me3 content than c-KIT–negative somatic cells at the early stage (Fig. 2c). Moreover, although female hPGCs exhibited even lower H3K27me3 signals, strong punctuated H3K27me3 signals could be still clearly observed in individual cells, which implied that it might play certain roles (Fig. 2d).

Moreover, despite their global low levels, these H3K27me3 signals were preferentially enriched at genes whose promoters were also occupied by H3K4me3 marks (Fig. 2e and Supplementary Fig. S2a). This subset of genes with markedly lower expression levels was indeed characterized by high H3K4me3 levels, similar to the bivalent state identified in hESCs (Fig. 2f and Supplementary Fig. S2c, d)^20^. For instance, the synaptonemal complex protein SYCP1 maintained a poised state in male hPGCs, whereas this gene lost its H3K27me3 marks and turned to an activated state in female hPGCs, in which it subsequently played a central role during meiotic prophase (Fig. 2g, h). Another important bivalent gene discovered in the hPGCs of both sexes was *TFAP2B*, whose expression was stimulated in preimplantation embryos but was switched to a poised state during PGC development (Supplementary Fig. S2e, f)^20^. Such a transcriptionally poised state is also widely observed in other systems, such as early embryo development and the mouse prenatal germline system, whereas the regulatory characteristics might be distinct among different stages and species^14,16^. Notably, we found that the total number of bivalent genes in hPGCs was much lower than that in the gonadal somatic cells or the hESCs (Fig. 2i and Supplementary Fig. S2g). Moreover, the dynamics of bivalent promoters in hPGCs mostly depended on the occupation or removal of H3K27me3 enrichment (Fig. 2j).

### Restriction of complete X chromosome reactivation in female hPGCs by repressive histone marks

**Fig. 3:**
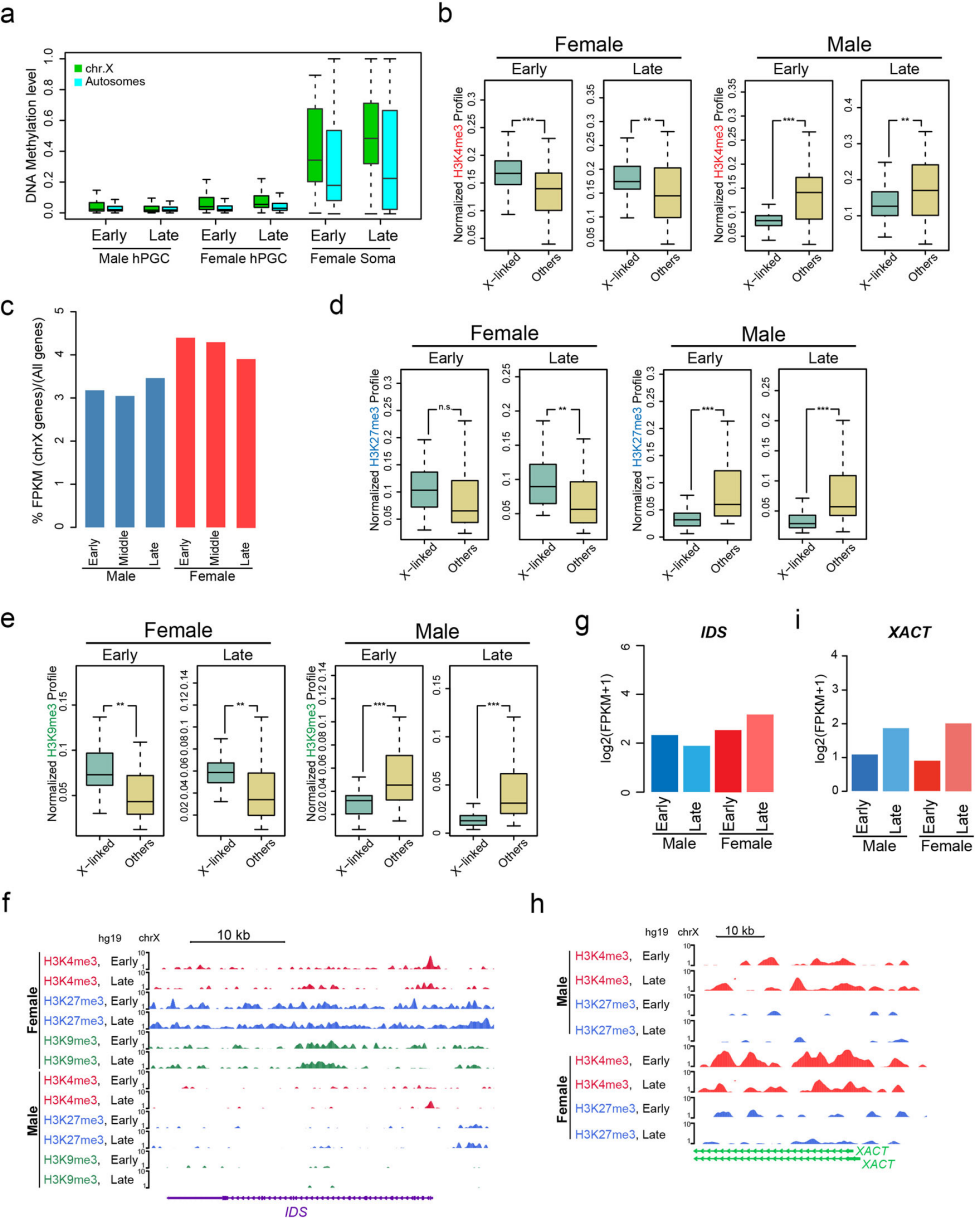
Regulation of X chromosome reactivation by histone modifications in female hPGCs. **a** Boxplot showing the comparison of DNA methylation levels at chrX gene (green) and other chromosome gene (bright blue) promoters in hPGC and gonadal somatic cell (Soma) samples. **b** Boxplots showing the comparison of H3K4me3 profiles on chrX CpG-island (CGI) promoters and other promoters in hPGCs of both sexes at early and late stage. Profiles were calculated as ChIP-seq RPM. **c** Barplot showing the proportion of total expression levels on chrX genes/all genes in male and female hPGCs at indicated stages. **d** Boxplots showing the comparison of H3K27me3 profiles on chrX CGI promoters and other promoters in hPGCs of both sexes at early and late stage. **e** Boxplots showing the comparison of H3K9me3 profiles on chrX CGI promoters and other promoters in hPGCs of both sexes at early and late stage. **f** Genome browser view of H3K4me3, H3K27me3 and H3K9me3 profiles on X reactivated gene *IDS*. Profiles were calculated as ChIP-seq RPM and smoothed as 3 pixels window using WashU Epigenome Browser. **g** Barplot showing *IDS* expression in hPGCs of both sexes at early and late stage. **h** Genome browser view of H3K4me3 and H3K27me3 profiles on lncRNA XACT. Profiles were calculated as ChIP-seq RPM and smoothed as 3 pixels window using WashU Epigenome Browser. **i** Barplot showing *XACT* expression in hPGCs of both sexes at early and late stage. For male, Wk8–9, Wk15 and Wk23 hPGC samples are represented as early, middle and late stage, respectively; for female, Wk8–10, Wk14 and Wk21 hPGC samples are represented as early, middle and late stage, respectively. CGI-promoters listed on chrX in (**b**, **d** and **e**) are cited from SRP057098. F female, M male. Student’s *t* test is performed to examine the significant statistical difference between two groups of data in boxplots in (**b**, **d** and **e**). **P < 0.01, ***P < 0.001, n.s. not significant.

X chromosome reactivation is one of the most significant phenomena occurring during female hPGC reprogramming. It has been indicated that X reactivation takes place prior to Wk4 and is maintained at least for 7 weeks in female fetal embryos^6^, despite the observation that XIST noncoding RNA is detectable^5^. Consistent with the observed genome-wide hypomethylation, we observed an extremely low trend of DNA methylation on X chromosomes, similar to that on the autosomes in female hPGCs (Fig. 3a). However, the minimal methylation level variations between female and male PGCs implied that this unique demethylated status might not fully account for the appropriate reactivation of X-inactivated alleles in female hPGCs (Fig. 3a). Here, we showed for the first time that the CGI promoters of X chromosomes were generally occupied by H3K4me3 signals compared to other randomly selected promoters, which was uniquely detected in female rather than male hPGCs (Fig. 3b). This result highly indicated that X chromosome reactivation in female hPGCs probably requires these active H3K4me3 marks.

However, an intriguing contradiction was also observed. Considering the global hypomethylation state and the enrichment of active H3K4me3 signals on the X chromosomes of female hPGCs, why did the activation of both X chromosomes cause only an ~1.6-fold increase in gene expression rather than the expected twofold increase^6^ (Fig. 3c)? To answer this question, we further examined whether certain repressive histone modifications or mechanisms were in place. Indeed, compared with male hPGCs at equivalent developmental stages, female hPGCs exhibited much higher H3K27me3 and H3K9me3 levels on the X chromosomes (Fig. 3d–g). This might partially account for the punctuated H3K27me3 fluorescent signals observed in individual female hPGCs despite the global lower H3K27me3 levels (Fig. 2d). However, neither the presence of XACT (function in XCD in hPGCs)^21^ nor XIST noncoding RNA could fully explain the restricted X-linked gene expression observed in female hPGCs, because active H3K4me3 signals at both regulation regions is not restricted to female, which further indicated a unique regulation of X chromosome during PGC development (Fig. 3h, i and Supplementary Fig. S3a, b).

Collectively, our data depict a complex regulatory mechanism of X reactivation during hPGC reprogramming in which full activation mediated by both DNA hypomethylation and H3K4me3 on two X chromosome are inhibited by repressive histone modifications. This subsequently leads to incomplete reactivation of the X chromosomes, as observed in female hPGCs.

### Histone modifications account for DNA demethylation escapees in hPGCs

Both male and female hPGCs are already hypomethylated in Wk4 during migration, and DNA methylation further declines to ~4% of the basal level around Wk10; this level is much lower than that in the inner cell mass (~37%) of human preimplantation embryos^10^. Interestingly, despite the global absence of 5mC in hPGCs, genomic regions that evaded hypomethylation could be identified, which can be simply divided into repeat-poor (2.3%) and repeat-rich (97.7%) escapees (Supplementary Fig. S3c)^10^. However, the mechanisms underlying demethylation resistance in a globally hypomethylated environment in the human germline have not been illuminated. Thus, we asked whether repressive histone marks might account for this contradiction.

**Fig. 4:**
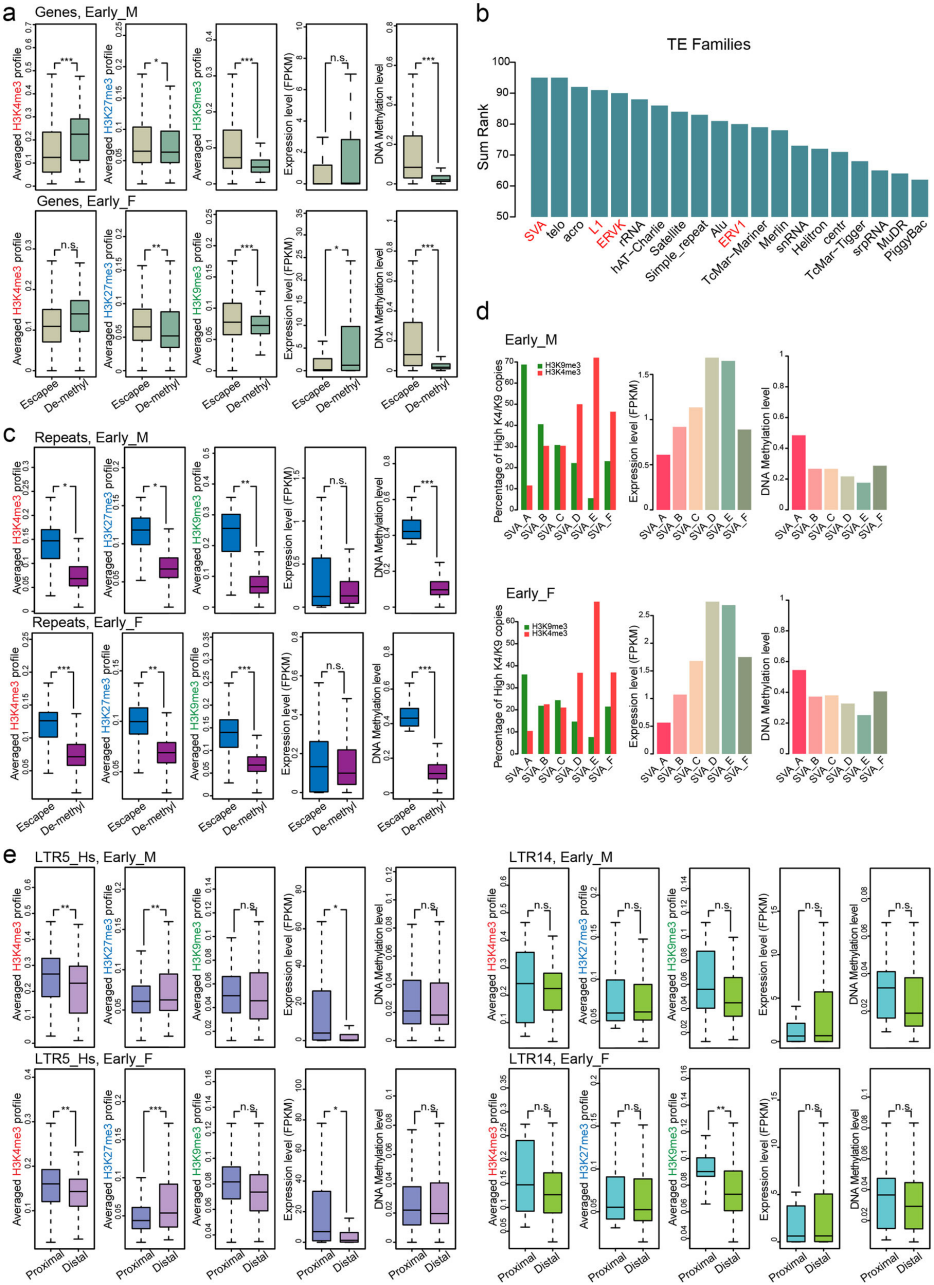
Chromatin state of DNA demethylation resistant loci in hPGCs. **a** Boxplots showing the comparison of H3K4me3, H3K27me3, H3K9me3 ChIP-seq profiles, expression (FPKM) and DNA methylation level between escapee and demethylated promoters in male and female hPGCs at early stage. **b** Barplot showing the sum of DNA methylation and expression level rank of retro-transposable elements in hPGCs. Higher rank indicates higher DNA methylation and expression level. SVA, LINE1(L1) and hominoid-restricted ERVs are indicated in red. **c** Boxplots showing the comparison of H3K4me3, H3K27me3, H3K9me3 ChIP-seq profiles, expression and DNA methylation levels between escapee and demethylated repeats in male and female hPGCs at early stage. **d** Barplots showing histone marks, expression and DNA methylation level in SVA subfamilies in male (top panel) and female (bottom panel) hPGCs at early stage. Left panel: barplot showing H3K4me3 and H3K9me3 preference of each copy of SVA subfamily. Copies with a higher (> 1.33 fold) H3K4me3 signal than H3K9me3 were defined as H3K4me3-marked, while lower (< 0.66 fold) ones were defined as H3K9me3-marked. Middle panel: barplot showing the expression level of each SVA subfamily. Right panel: barplot showing the DNA methylation level of each SVA subfamily. **e** Boxplots showing the comparison of H3K4me3, H3K27me3, H3K9me3 ChIP-seq profiles, expression levels (FPKM) and DNA methylation levels between proximal and distal genes relative to LTR5_Hs/HERVK subfamily (left panel) and LTR14/HERVK subfamily (right panel) in male (top panel) and female (bottom panel) hPGCs at early stage. For male, Wk10 and Wk23 hPGC samples are represented as early and late stage, respectively; for female, Wk10 and Wk21 hPGC samples are represented as early and late stage, respectively. F female, M male, De-methyl demethylated. Student’s *t* test is performed to examine the significant statistical difference between two groups of data in boxplots in (**a**, **c** and **e**). *P < 0.05, **P < 0.01, ***P < 0.001, n.s. not significant.

We first assessed the epigenetic landscapes of demethylation-resistant genes and their regulatory regions. In line with our expectation, H3K9me3 was shown to primarily mark these escapee regions in vivo rather than their demethylated counterparts (Fig. 4a). In addition, H3K27me3 potentially serves as a consolidator for these relatively hypermethylated regions in hPGCs (Fig. 4a). Notably, repressive H3K9me3 and H3K27me3 marks were detected in these demethylation-resistant genes (such as *STX2*) as early as Wk8 (Fig. 4a and Supplementary Fig. S3d, e), suggesting that these two marks were sufficient to safeguard these escapee genes during the later course of germline development, since DNA methylation continued to decline thereafter. Moreover, the persistent enrichment of H3K9me3 and H3K27me3 at escapee promoters was generally compatible with the residual hyper-DNA methylation marks observed in hPGCs, which further indicates a synergistic effect of hierarchical epigenetic modifications in transcriptional repression during hPGC development (Fig. 4a). As a comparison, demethylated genes rarely presented both H3K9me3 and H3K27me3 marks and showed relative higher expression level (Fig. 4a and Supplementary Fig. S3d, e). In addition, these H3K9me3 and H3K27me3 signals were retained in the repeat-poor escapees of hPGCs for more than 10 weeks, as both were detected in Wk20+ hPGCs (Supplementary Fig. S3f).

With the exception of the repeat-poor escapees, up to 97.7% of the regions evading genome-wide DNA demethylation in hPGCs were predominantly covered by retrotransposon elements (Supplementary Fig. S3c). Intriguingly, the DNA hypermethylation status of particular retrotransposons was not consistently accompanied by their global transcriptional repression in hPGCs. Instead, the majority of evolutionarily younger and currently active transposons, such as the hominoid-restricted ERVK (HERVK) and SVA families, displayed a significant positive correlation between methylation and expression compared to their older counterparts (Fig. 4b and Supplementary Fig. S4a)^5,6,10^. Thus, these data suggested that additional epigenetic regulators may help to orchestrate the appropriate transcription of these repeat-rich escapees over such a long period during hPGC development. Strikingly, unlike the relative less H3K4me3 signals in escapee promoters (Fig. 4a and Supplementary Fig. S3f), hypermethylated repeat-rich escapees were sufficiently marked by both active (H3K4me3) and repressive (H3K9me3 and H3K27me3) chromatin marks (Fig. 4c and Supplementary Fig. S4b). Thus, it was of great interest for us to finely examine these repeat-rich escapee regions.

We then focused on SVAs, which showed an obviously higher methylation level across all hPGC development stages in both sexes but were also activated (Fig. 4b and Supplementary Fig. S4a)^5,10^. Indeed, we observed that active and repressive histone marks were distributed separately among different SVA subfamilies (Supplementary Fig. S4c). For example, H3K9me3 marks were relatively prominently enriched in the SVA_A and SVA_B subfamilies, while SVA_D and SVA_E subfamilies were preferentially marked by H3K4me3 with lower H3K9me9 enrichment (Fig. 4d and Supplementary Fig. S4c, d). This kind of fine-tuning led to the activation of the SVA_D and SVA_E subfamilies and ultimately contributed to a lack of prominent derepression of SVAs in hPGCs^10^ (Supplementary Fig. S4a).

Active repeat-rich escapees may impact the transcription of their surrounding genomic regions, which can be referred to as in vivo positional effects. According to recently annotated enhancers embedded in younger transposons in the human preimplantation embryonic genome, these transposable element-based enhancers (TEs) could be introduced in proximal regulatory sequences, where they might rapidly stimulate the transcriptional activity of these regions^22^. To address the possible causes of the presence of these TEs in the human germline, we explored the chromatin state and corresponding activities of genes situated in the vicinity of such activated TEs in hPGCs. We observed that active TEs, including LTR5_Hs/HERVK, tended to activate local chromatin, as indicated by an increase in the active histone marker H3K4me3 and a decrease in the repressive histone marker H3K27me3. Meanwhile, DNA methylation and H3K9me3 showed no obvious differences (Fig. 4e and Supplementary Fig. S4e, f). As a result, neighboring gene expression appeared to be much higher than distal gene expression (Fig. 4e and Supplementary Fig. S4f, g). In contrast, either proximal or distal regions relative to the inactive LTR14/HERVK subfamily were consistently transcriptionally silenced in hPGCs, which was largely consistent with the similar chromatin state in both regions (Fig. 4e and Supplementary Fig. S4e–g). Overall, this result indicates the activation effect of intrinsic histone modifications on transposable element-based enhancers plays an essential role in facilitating local chromatin remodeling and consequently regulates the expression of proximal genes.

## Discussion

In this work, we present the first time-course profiles of three key histone modifications in the human germline. The core histone modification reference map obtained by using ULI-NChIP-seq from PGCs across multiple stages of human development provided insights into chromatin reorganization and cell fate regulation during hPGC development. Here, we demonstrate that the H3K4me3 distribution in hPGCs is insufficient compared to that in corresponding gonadal somatic cells, although these marks are still specifically located in key promoters and regulate the expression of related development- and transcription-associated genes. H3K27me3, however, mainly serves as a repressive regulator and coordinates with H3K4me3 to govern specific bivalent promoter regions. Notably, the unique, bewildering pattern of histone reprogramming throughout human germline development might be the cause of the transcriptionally flexible state observed in germ cells. Moreover, under the activation effects mediated by global DNA demethylation and H3K4me3 enrichment, repressive H3K27me3 and H3K9me3 marks jointly inhibit the full activation of X chromosomes in female hPGCs. In addition, we identified a dual role of H3K9me3 in not only protecting the genomic stability of globally demethylated regions but also preventing methylation from being removed in demethylation-resistant regions in human prenatal germline.

The overall DNA demethylation dynamics in hPGCs are similar to those in the mouse germline, with DNA methylation decreasing to the exceptionally low levels when migratory PGCs of both species settle into the genital ridge^5,6,10,23^. However, methylation is then re­established in a sex­-specific manner, before birth in males and after birth in females^24–26^. Previous studies have demonstrated that the extreme genome-wide hypomethylation does not lead to excessive transcriptional excitability in the human germline^5,10^. Thus, how hPGCs maintain a relatively unexcitable transcriptome when DNA methylation is globally reduced remains a mystery. Do the heterochromatin-related histone modifications (such as H3K27me3 and H3K9me3) play roles in suppressing gene expression so as to compensate DNA hypomethylation? Or do the active histone modifications (such as H3K4me3) show a depleted state so that genes and non-genes could not be activated? These questions triggered us great interests. Here, we presented a complex interaction network of three histone modifications (H3K4me3, H3K27me3 and H3K9me3) and DNA methylation, which cooperatively regulate gene and non-gene expression, as well as multiple key events during hPGC development such as X chromosome reactivation and DNA demethylation escapees.

In addition, we demonstrated that although both sexes showed persistently low H3K27me3 and H3K9me3 signals, active H3K4me3 marks also exhibited lower abundance than in gonadal somatic cells, which indicates the occurrence of major changes in the nuclear architecture accompanied by the extensive erasure of certain core histone modifications during human germ cell development. This unique chaotic state involving relatively weak epigenetic enrichment and highly open chromatin indicates the occurrence of extensive reprogramming in the human germline, which provides a flexible state for receiving external signals and completing gametogenesis^9^. Moreover, the maintenance of such a transcriptionally flexible state in the germ cells might facilitate reprogramming to totipotency following fertilization. And the molecular mechanism underlying the global loss of DNA methylation that triggers the reorganization of chromatin modifications to modulate gene or retrotransposon expression and safeguard genome integrity has been previously revealed in mouse PGCs^15,17,19,27^.

Broad H3K4me3 domains exceeding 5 kb around the TSS are generally observed in mouse peri-implantation embryos and are associated with high transcription and cell identity^18,28^. However, in contrast to the typical broad H3K4me3 peaks, most promoter enriched H3K4me3 signals cover only a 1–2 kb region around the TSS in both human and mouse^14–16^ PGCs. In addition, a recent study demonstrated that broad H3K4me3 domains are not observable in human early embryos^20^. Above all, these results indicate that the broad H3K4me3 pattern may be a unique feature that ensures transcriptional precision at key cell identity/function-related genes in mouse early embryo development but not in hPGC and human preimplantation embryo development.

Cellular heterogeneity is a critical obstacle in the study of complex prenatal germ cell lineage systems in humans^29^. Since key developmental processes are often initiated in small populations of cells, our ULI-NChIP-seq-based method was unable to map all these three histone modifications (H3K4me3, H3K27me3 and H3K9me3) in an individual embryo in all cases, particularly in very early stages. Achieving such a consistent map would require the further improvement of existing epigenome technologies, and our understanding of the different layers of epigenetic regulation in human germline development could be greatly increased through the comprehensive multiomics analyses including data on histone modifications, DNA methylation and RNA transcription within an individual embryo. However, high gene expression heterogeneity has been reported among individual hPGCs, even though originating from the same embryo^6,9^. Moreover, due to the scarcity of hPGCs, the elucidation of genomic occupancy of transcription factors has been impeded thus far. Because of the important roles of rare cell populations in each developmental stage, more sensitive methods such as recently described CUT (cleavage under targets)-based approaches for very low cell numbers or even single cells might help to advance the characterization of epigenetic regulation and functions in human prenatal germline development^30–32^.

In conclusion, we provide a unique roadmap of three core histone modifications during hPGC development, which helps to elucidate the architecture of germ cell reprogramming in a DNA–hypomethylated environment.

## Materials and methods

### Collection of human fetal samples

The donors in this study were pregnant women who underwent medical termination of pregnancy (due to conditions such as cervical insufficiency, inevitable abortion, eclampsia, etc.). All of the patients signed informed consents and voluntarily donated the fetal tissues for this study. The human embryos in this study were from aborted fetuses at 8–23 weeks of gestation, and the stages of all samples were calculated from the last menstruation bleeding time. The experiments performed in this study were approved by the Reproductive Study Ethics Committee of Shanghai First Maternity and Infant Hospital, and the approved number is KS1888. In total, we collected 8 male embryos and 5 female embryos for sequencing in this study, and the detailed information of these samples were listed in Supplementary Fig. S1a. Besides, we also collected 2 male embryos and 3 female embryos for Immunofluorescence analysis or Reverse Transcription and quantitative Real-Time PCR (RT-qPCR) validation.

Identification of externalia phenotype and genotyping were combined for sex determination of each collected embryo between 8- and 23-week. For genotyping, the genomic DNA was lysed by KAPA Express Extract Kit (KK7103, KAPA Biosystems) and used for Y chromosome genotyping (*TSPY2* gene and *SRY* gene). The *CCR6* gene on chromosome 6 was included as a control for both male and female embryos. Three primer pairs used for genotyping are listed below:

*CCR6*-F: 5′-GGAATATGGGGCAAAGGACA-3′

*CCR6*-R: 5′-GGCTGGTTGCCTTTACTTCG-3′

*TSPY2*-F: 5′-GGGCCAATGTTGTATCCTTCTC-3′

*TSPY2*-R: 5′-GCCCATCGGTCACTTACACTTC-3′

*SRY*-F: 5′-CCAGAAGTGAGCCTGCCTAT-3′

*SRY*-R: 5′-GACTGCTTAACACGCTGCAT-3′

### Isolation of human fetal PGCs and gonadal somatic cells by FACS

For 8–23-week human embryos, the gonads were dissected in DPBS (plus 10% FBS) and separated from surrounding mesonephric tissues. A small amount of tissue from the fetus would be collected for gender testing by PCR as described above. The gonads were further digested by using Collagenase/Dispase (Sigma) for 5–15 min at 37 °C (depending on the size of the gonad) to dissociate into single cell suspension and labeled by PE mouse anti-human CD117 (BD, #555714, clone YB5.B8, also known as C-KIT) as previously described^6^. Then CD117-postive hPGCs and CD117-negative gonadal somatic cells could be isolated by BD FACS AriaII. In each experiment, the same sample with same treatment but without CD117 staining should be conducted and served as a negative control. Notably, erythrocytes in CD117-negative cells were further removed by using red blood cell lysis buffer (Tiangen) before ChIP-seq and RNA-seq.

### Immunofluorescent staining

Human embryonic tissues were fixed in 4% paraformaldehyde for 2 h at 4 °C. Fixed tissues were prepared as 8 μm cryosections and then immunofluorescent staining was performed as previously described^33^. Briefly, all samples were incubated with primary antibodies overnight at 4 °C (Oct4, Santa Cruz sc-5279; c-KIT, BD #555714, clone YB5.B8; H3K4me3, CST #9727; H3K27me3, Diagnode C15410069). Sections were washed, incubated with AF488/AF594/AF633 conjugated secondary antibodies (Invitrogen) for 45 min at room temperature and mounted in prolong anti-fade reagent with DAPI (Sigma). Confocal imaging was performed with Zeiss LSM 880 confocal microscopes and analyzed with Zeiss Zen blue edition.

### ULI-NChIP-seq

The ULI-NChIP procedure was performed as previously described^34^. Approximately 300–1000 hPGCs or somatic cells were used per reaction. The libraries were generated using the KAPA Hyper Prep Kit according to the manufacturer’s instructions, and sequenced on an Illumina Hiseq X Ten with a paired-end 150 bp protocol at the Berry Genomics Co., Ltd.

### Smart-seq2

For Smart-seq2, ~50 hPGCs or somatic cells were used per reaction. RNA-seq libraries were generated using the Covaris DNA shearing protocol for Smart-seq sequence library generation as previously described^35^. Briefly, RNAs with a poly-adenylated tail were captured, reverse transcribed and pre-amplified. After fragmentation, the sequence libraries were generated by using the KAPA Hyper Prep Kit for the Illumina platform, following the manufacturer’s instructions. Paired-end 150 bp sequencing was performed on Hiseq (Illumina) platform at the Berry Genomics Co., Ltd.

### RT-qPCR

PGCs or somatic cells were disrupted in TRIzol Reagent (Takara) and total RNAs were isolated by chloroform extraction coupled with isopropanol precipitation, with 1/10 volume of 3 mol/L NaAc and 1 μL glycogen was added to the aqueous phase of each sample. RNAs were then washed twice with 75% ethanol before they were eluted with nuclease-free water. cDNA was then synthesized using All-In-One RT MasterMix (Applied Biological Materials). qPCR was carried out using TB Green Premix Ex Taq II (Takara Bio) and monitored by 7500 Fast Real-Time PCR System, and three technical replicates were performed for each sample. Relative expression level of each gene was normalized to the reference gene *GAPDH*. qPCR primers for tested genes are listed in Supplementary Table S1.

### ChIP-seq, RNA-seq, DNase-seq and WGBS data processing

For human samples, ChIP-seq reads were aligned to the human genome (hg19 assembly) using bowtie2^36^ version 2.2.9 with default parameters. MACS (v1.4.2)^37^ was used to call peaks from mapped reads of each sample by callpeak function with parameters -nomodel -shiftsize 73. Signal tracks were generated by MACS2 (v2.1.1) pileup function and normalized to 1 million reads for profiling and visualization with parameters -nomodel -shift 73 –SPMR. We calculated the normalized ChIP-seq signal profile correlation on all RefSeq annotated gene promoters (defined as ± 2 kb of TSS) of hg19 genome build to examine the reproducibility of our ChIP-seq experiments. Qualified biological replicates were pooled together for downstream analysis. 8–10 week samples were defined as early stage, 11–15 week samples were defined as middle stage, and 20–23 week samples were defined as late stage for downstream.

Reads of hPGCLC were download from GSE159654^38^ and processed by the same workflow, except signal tracks were pileup by BEDPE mode. Reads of mPGC were downloaded from SRA097278^14^, mapped to the mouse genome (mm9 assembly), and processed like above.

The RNA-seq reads were mapped to human genome (hg19 assembly) using TopHat (v2.1.1)^39^. Expression levels were quantified as FPKM using Cufflinks (v2.1.1)^40^. FPKM values from qualified biological replicates were averaged for downstream analysis. Reads were mapped by STAR (v2.5.2b)^41^ and quantified as FPKM by StringTie (v2.1.5)^42^ for the expression of XIST and XACT in particular.

The DNase-seq reads from GSE109768^8^ were trimmed by fastp (v0.23.2)^43^ and mapped to human genome (hg19 assembly) using bowtie2. Replicates were merged to generate signal tracks by MACS2(v2.1.1) callpeak with parameters -B –SPMR.

We took advantage of published WGBS data in hPGC from GSE63818^6^. For DNA methylation of early female soma cells, raw reads were downloaded from SRR1777327^10^ and mapped to human genome (hg19 assembly) by bsmap (v2.90)^44^ and methylation levels were called by mcall (v1.0)^45^. The methylation level of each CpG site and methylation signal tracks were generated in downstream analysis.

### Expression level quantification on transposable elements

All RNA-seq reads were re-mapped to human genome (hg19 assembly) using STAR aligner^41^ with parameters –outFilterMismatchNmax 3 –outFilterMultimapNmax 500 to tolerate mapping mismatches on genome and filter out reads mapping to more than 500 locations on genome. Tag directories were generated using HOMER^46^ makeTagDirectory function with parameter -keepOne from mapped reads. Then analyzeRepeats.pl script in HOMER was used to analyze tag directories with repeat function and parameter -noadj and summarize multiple mapped tags to their representative TE families. Total counts were normalized to 1 million on each TE family and averaged among biological replicates for further analysis. For each TE sub-family, we defined union set of genes within ± 10 kb of a certain annotated copy as sub-family ‘around genes’. By contrast, other genes were ‘distal genes’ of this TE sub-family.

Repeat element annotation of hg19 genome was downloaded from UCSC table browser^47^, and we re-calculated ChIP-seq and DNA methylation signal profiles on each TE copy and then signal tracks were generated.

### Definition of demethylation escapees

We divided the human genome (assembly hg19) into non-overlapping 5 kb bins and took the mean DNA methylation levels during PGC development as the average DNA methylation level. Promoters in bins with an average DNA methylation level > 0.4 were defined as escapees. For transposable elements, subfamilies with mean DNA methylation level ≥ 0.35 were defined as escapees.

### Genomic enrichment calculation

RefSeq gene and repeats (assembly hg19) were downloaded from UCSC table browser (http://genome.ucsc.edu/cgi-bin/hgTables). The enrichment of ChIP-seq peaks of each sample on genomic regions was calculated as observed ratio versus expected ratio, which were defined as:

$$R_{observed} = \frac{{L_{peak\,overlapped\,with\,genomic\,region}}}{{L_{total\,peak}}}$$

$$R_{expected} = \frac{{L_{total\,genomic\,region}}}{{L_{total\,genome}}}$$

Then the peak enrichment on a certain genomic region could be calculated as:

$$E = \frac{{R_{observed}}}{{R_{expected}}}$$

Genes with promoters (TSS ± 2000 bp) covered by H3K4me3 or H3K27me3 peaks in each sample were designated as target genes and used in Gene Ontology analysis with g:Profiler^48^. Terms with fewer than 100 targets or more than 1000 targets were removed from the analysis. Sorted by adjusted *P* value, the top 20 terms were present in the Supplementary Table S2.

### Track visualization

Custom track hubs were uploaded to the WashU Epigenome Browser^49^ to visualize the signal tracks of ChIP-seq and DNA methylation data. For ChIP-seq data, data range was set from onefold to tenfold based on average signal of each sample. All tracks were smoothed based on mean signal of 3 pixels for visualization. For DNA methylation data, data range was set to 0–1.

## Supplementary information

Supplementary Figures S1–S4

Supplementary Table S1

Supplementary Table S2

## Data Availability

The raw sequence data reported in this paper have been deposited in the Genome Sequence Archive^50^ in National Genomics Data Center^51^, Beijing Institute of Genomics (China National Center for Bioinformation), Chinese Academy of Sciences, under accession number HRA000234 that are publicly accessible at https://bigd.big.ac.cn/gsa.
