# Supplementary material for: Resetting histone modifications during human prenatal germline development
Source: Cell Discov. 2023 Feb 3;9:14. doi: 10.1038/s41421-023-00519-1 (PMC9898496; doi:10.1038/s41421-023-00519-1)
Supplement: Supplementary file 1 — Supplementary Figures S1–S4 [file 41421_2023_519_MOESM1_ESM.pdf]

Supplementary Information for

# Resetting Histone Modifications During Human Prenatal Germline Development

**Rui Gao[1,4], Shiyang Zeng[2,4], Dongxu Yang[1,2,4], Xiaocui Li[1,4], Wenqiang Liu[1], Yawei Gao[2], Dandan Bai[1], Linfeng Zhang[1], Chuan Chen[1], Yunzhe Kang[1], Beiying Wang[1], Wei Hong[1], Mingzhu Wang[1], Jiqing Yin[1], Hong Wang[1], Qiaolin Deng[3], Shaorong Gao[1,2,*], Yong Zhang[2,*], Jiayu Chen[1,*].**

[1]Clinical and Translation Research Center of Shanghai First Maternity & Infant Hospital, Shanghai Key Laboratory of Signaling and Disease Research, Frontier Science Center for Stem Cell Research, School of Life Sciences and Technology, Tongji University, Shanghai 200092, China
[2]Institute for Regenerative Medicine, Shanghai East Hospital, Shanghai Key Laboratory of Signaling and Disease Research, Frontier Science Center for Stem Cell Research, School of Life Sciences and Technology, Tongji University, Shanghai, China.
[3]Dept. Physiology and pharmacology, Biomedicum B5, Karolinska Institutet, Center for Molecular Medicine, Karolinska University Hospital, Stockholm, Sweden.
[4]These authors contributed equally to this work.

*Correspondence: Jiayu Chen (chenjiayu@tongji.edu.cn), Yong Zhang (yzhang@tongji.edu.cn) or Shaorong Gao (gaoshaorong@tongji.edu.cn).

## This PDF file includes:

Supplementary Figures

Supplementary Figure Legends.

A

| Sample ID | Age (Week) | Stage | Gender | No. of Sample Replicate (hPGC / Soma) | | | |
|---|---|---|---|---|---|---|---|
| | | | | H3K4me3 ChIP-seq | H3K27me3 ChIP-seq | H3K9me3 ChIP-seq | RNA-seq |
| HRI043519 | 8 | Early | Male | 1 / 0 | 1 / 0 | 0 / 0 | 3 / 0 |
| HRI043520 | 9 | | | 0 / 0 | 0 / 0 | 1 / 0 | 0 / 0 |
| HRI043521 | 10 | | | 2 / 1 | 2 / 1 | 0 / 0 | 0 / 0 |
| HRI043531 | 11 | Middle | | 0 / 0 | 0 / 0 | 0 / 0 | 3 / 3 |
| HRI043522 | 12 | | | 2 / 0 | 2 / 0 | 0 / 0 | 0 / 0 |
| HRI043523 | 15 | | | 2 / 2 | 2 / 2 | 2 / 2 | 3 / 3 |
| HRI043524 | 21 | Late | | 2 / 2 | 2 / 2 | 2 / 0 | 0 / 0 |
| HRI043525 | 23 | | | 2 / 0 | 2 / 0 | 2 / 2 | 3 / 3 |
| Total | - | - | Male | 11 / 5 | 11 / 5 | 7 / 0 | 12 / 9 |
| HRI043526 | 8 | Early | Female | 1 / 0 | 1 / 0 | 1 / 1 | 2 / 0 |
| HRI043527 | 10 | | | 2 / 1 | 2 / 1 | 0 / 1 | 0 / 0 |
| HRI043528 | 14 | Middle | | 2 / 0 | 2 / 0 | 0 / 1 | 3 / 3 |
| HRI043529 | 15 | | | 2 / 0 | 2 / 0 | 0 / 0 | 0 / 0 |
| HRI043530 | 21 | Late | | 1 / 2 | 2 / 2 | 2 / 2 | 3 / 3 |
| Total | - | - | Female | 8 / 3 | 9 / 3 | 3 / 5 | 8 / 6 |

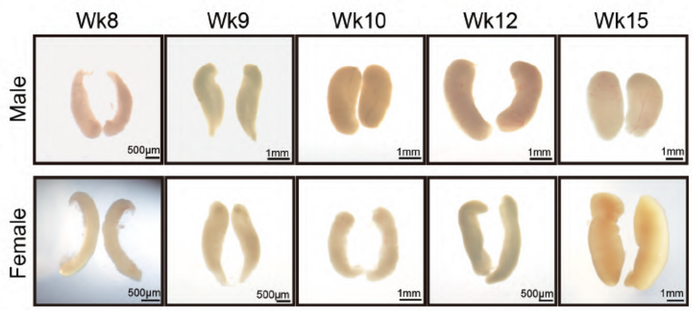


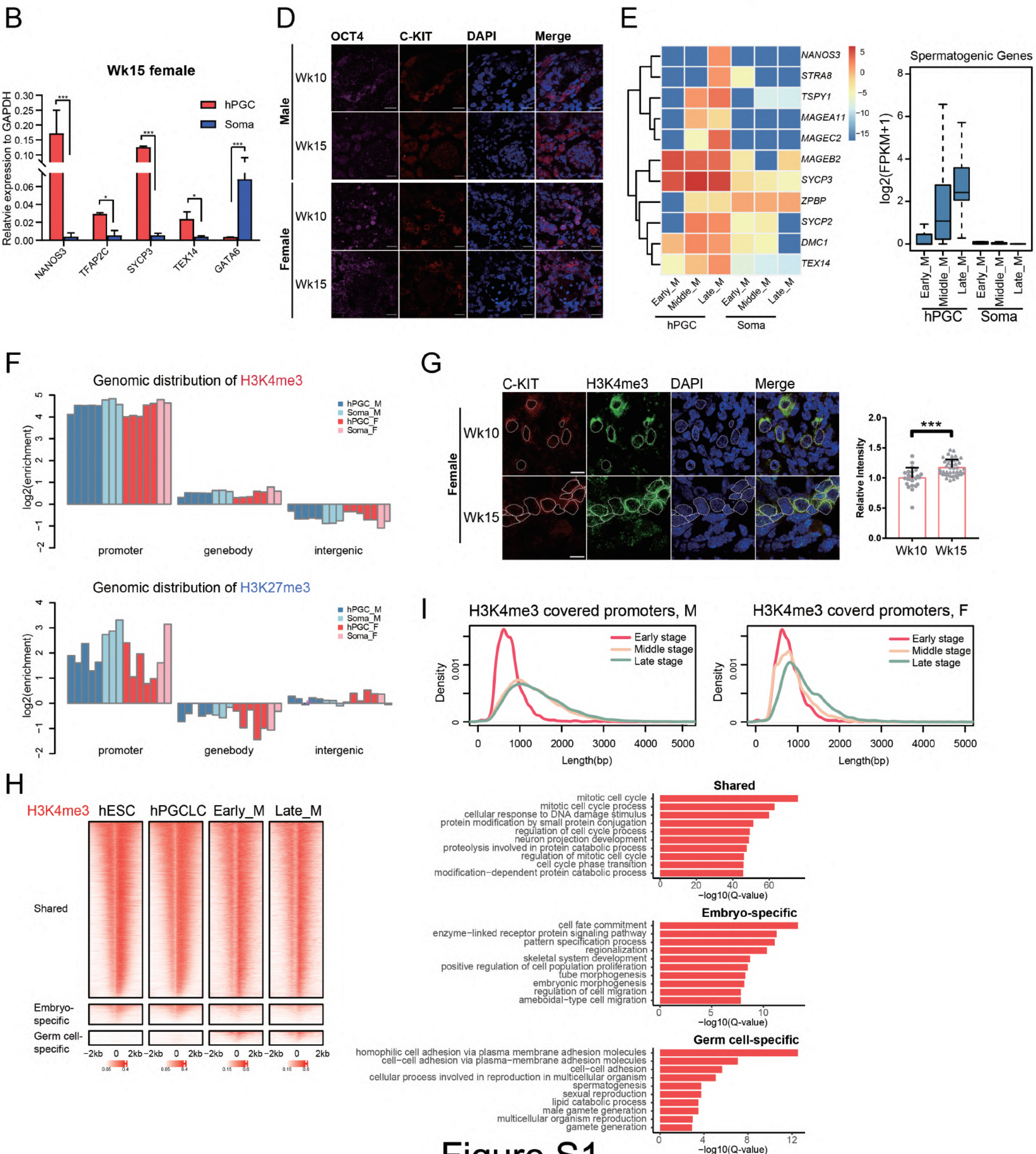


Figure S1

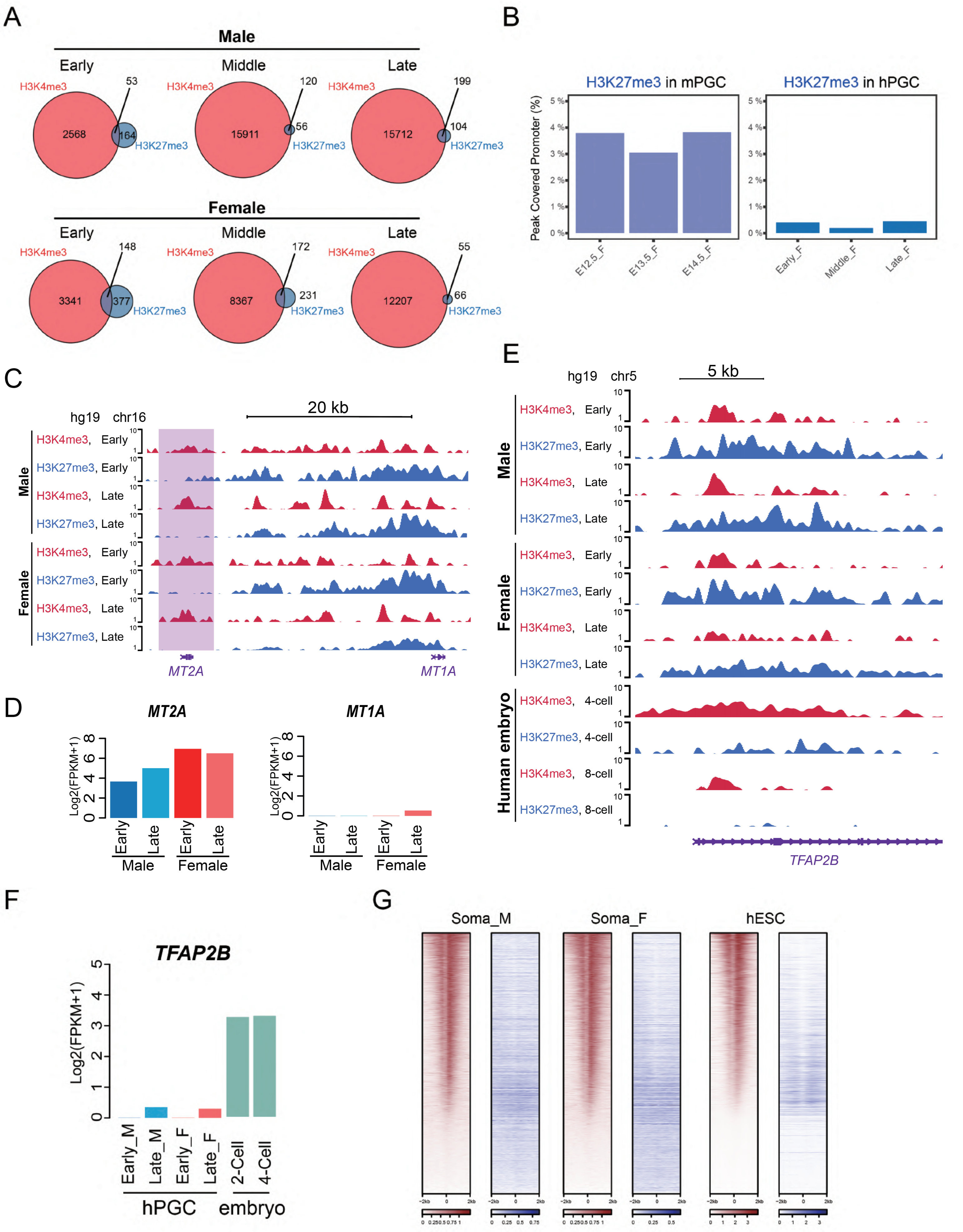
A
Male
Early
Middle
Late
H3K4me3
H3K27me3
53
2568
164
120
15911
56
199
15712
104
Female
148
3341
377
172
8367
231
55
12207
66
B
H3K27me3 in mPGC
H3K27me3 in hPGC
Peak Covered Promoter (%)
E12.5_F
E13.5_F
E14.5_F
Early_F
Middle_F
Late_F
C
hg19 chr16
20 kb
MT2A
MT1A
D
MT2A
MT1A
Log2(FPKM+1)
E
hg19 chr5
5 kb
Human embryo
4-cell
8-cell
TFAP2B
F
TFAP2B
Early_M
Late_M
Early_F
Late_F
2-Cell
4-Cell
hPGC
embryo
G
Soma_M
Soma_F
hESC

Figure S2

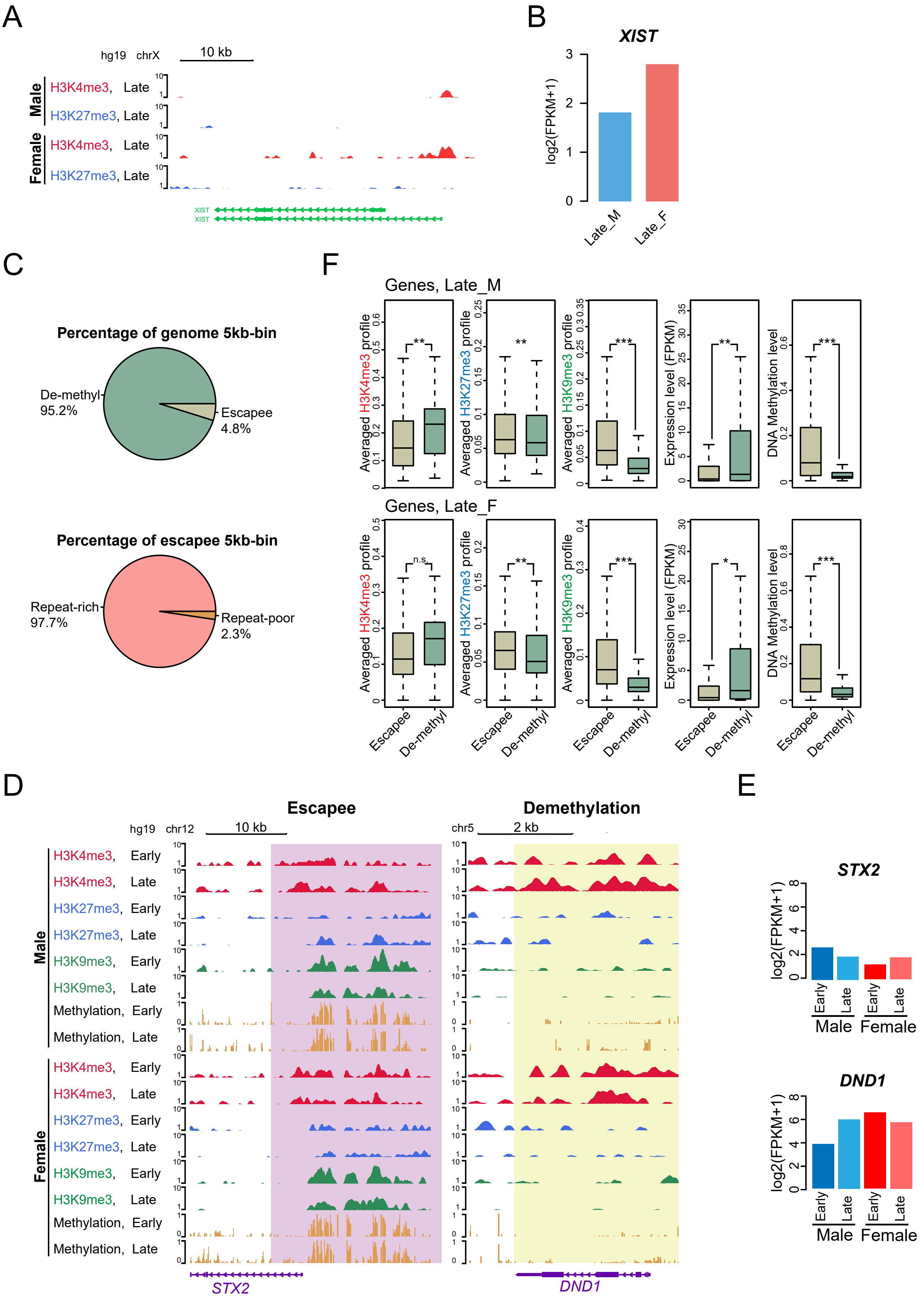

A
hg19 chrX
10 kb
Male
Female
H3K4me3, Late
H3K27me3, Late
XIST
B
XIST
log2(FPKM+1)
Late_M
Late_F
C
Percentage of genome 5kb-bin
De-methyl 95.2%
Escapee 4.8%
Percentage of escapee 5kb-bin
Repeat-rich 97.7%
Repeat-poor 2.3%
F
Genes, Late_M
Genes, Late_F
Averaged H3K4me3 profile
Averaged H3K27me3 profile
Averaged H3K9me3 profile
Expression level (FPKM)
DNA Methylation level
Escapee
De-methyl
n.s.
D
Escapee
Demethylation
hg19 chr12
10 kb
chr5
2 kb
H3K4me3, Early
H3K4me3, Late
H3K27me3, Early
H3K27me3, Late
H3K9me3, Early
H3K9me3, Late
Methylation, Early
Methylation, Late
STX2
DND1
E
STX2
DND1
log2(FPKM+1)
Early
Late
Male
Female


Figure S3

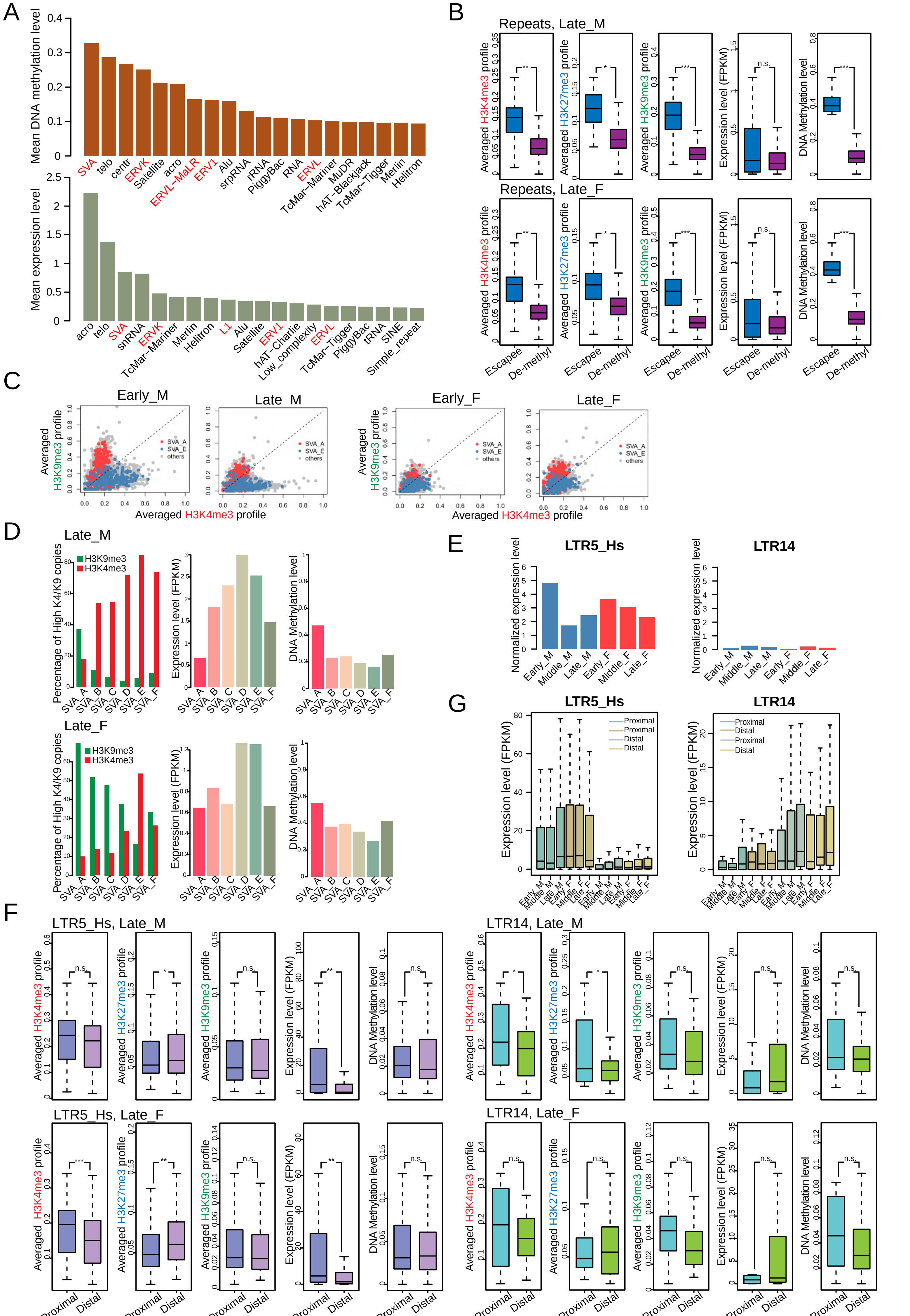

A
Mean DNA methylation level
Mean expression level
B
Repeats, Late_M
Repeats, Late_F
Escapee
De-methyl
C
Early_M
Late_M
Early_F
Late_F
Averaged H3K9me3 profile
Averaged H3K4me3 profile
SVA_A
SVA_E
others
D
Late_M
Late_F
Percentage of High K4/K9 copies
H3K9me3
H3K4me3
Expression level (FPKM)
DNA Methylation level
E
LTR5_Hs
LTR14
Normalized expression level
G
LTR5_Hs
LTR14
Proximal
Distal
F
LTR5_Hs, Late_M
LTR14, Late_M
LTR5_Hs, Late_F
LTR14, Late_F
Proximal
Distal

Figure S4

**Supplementary Figure legends**

**Figure S1, related to Figure 1, Dynamics of H3K4me3 and H3K27me3 chromatin domains in hPGCs**

(A) Summary of hPGCs and gonadal somatic cells analyzed by ULI-NChIP and RNA-seq techniques.

(B) RT-qPCR analysis of selected marker genes in hPGCs and somatic cells in a 15-week (Wk15) female sample.

(C) Morphology of Wk8 to Wk15 human genital ridges of both sexes.

(D) Representative immunofluorescence micrographs of OCT4 and c-KIT in genital ridge cryosections at Wk10 and Wk15 of both sexes. Scale bars, 20 μm.

(E) Heatmap (left) and boxplot (right) for representative spermatogenic genes expression in male hPGC and gonadal somatic cell (Soma) samples at indicated development stages. Expression was present as log2(1e-6 + FPKM) and log2(FPKM+1) in boxplot.

(F) Barplots showing the genomic distributions of H3K4me3 and H3K27me3 ChIP-seq peaks on promoters, genebody and intergenic regions in hPGCs in indicated samples of both sexes. Soma, gonadal somatic cells.

(G) (left) Immunofluorescence analysis for H3K4me3 on Wk10 and Wk15 female genital ridge cryosections. The nuclei of c-KIT positive hPGCs are indicated by white dotted line. Scale bars, 10 μm. (right) Analysis of relative intensity of H3K4me3 of c-KIT positive female hPGCs between Wk10 and Wk15. The results are normalized to those in Wk10. Each dot represents the relative fluorescence intensity of a single nucleus. Data are represented as the means ± SEM ($n \geq 23$ single nuclei).

(H) Heatmaps showing the distribution of H3K4me3 profiles around TSS (±2 kb) in hPGCLC, hESC and hPGC samples. The right panels show the enriched GO terms of biological processes with the q-values. The difference between H3K4me3 peaks was used to define shared, embryo-specific, and germ cell-specific genes using the following rules: shared: the promoter was covered in hESC, hPGCLC, and at least

one hPGC sample; embryo-specific: the promoter was covered in hESC or hPGCLC but not any hPGC samples; germ cell-specific: the promoter was covered in hPGCLC or at least one hPGC sample but not hESC.

(I) Line plots showing the density distribution dynamics of H3K4me3 covered promoters in male and female hPGC samples at indicated stages.

In Figure S1, for male, Wk8-10, Wk12-15 and Wk23 hPGC samples are represented as early, middle and late stage, respectively; for female, Wk8, Wk14 and Wk21 hPGC samples are represented as early, middle and late stage, respectively. Soma, gonadal somatic cells; F, female; M, male.

**Figure S2, related to Figure 2, Bivalent chromatin domains in hPGCs**

(A) Venn diagrams showing the number of H3K4me3 and H3K27me3 ChIP-seq peak covered promoters at early, middle and late stage in male and female hPGC samples.

(B) Barplots showing the percentage of H3K27me3 covered promoters in female mPGC (left) and female hPGC (right) samples. H3K27me3 profiles of mouse PGCs are cited from SRA097278.

(C) Genome browser view of H3K4me3 and H3K27me3 profiles on H3K4me3-only gene *MT2A* (shaded in light purple) and bivalent gene *MT1A*. Profiles are calculated as ChIP-seq RPM and smoothed as 3 pixels window using WashU Epigenome Browser. Data range is set as 1-fold to 10-fold of average RPM in each sample.

(D) Barplot showing *MT2A* and *MT1A* expression in hPGCs of both sexes at early and late stage.

(E) Genome browser view of H3K4me3 and H3K27me3 profiles at bivalent gene *TFAP2B* locus in hPGCs of both sexes at indicated time point. The histone profiles at *TFAP2B* locus in human pre-implanted embryos at 4-cell and 8-cell stages are also indicated. H3K4me3 and H3K27me3 profiles and expression levels of human pre-implanted embryos are cited from GSE124718.

(F) Barplot showing *TFAP2B* expression in hPGCs and human pre-implanted embryos at indicated stages.

(G) Heatmaps showing the distribution of H3K4me3 and H3K27me3 profiles around

TSS (±2kb) in gonadal somatic cell samples of both sexes and human ESCs. Data of human ESCs is cited from GSE29611. Signals are sorted by H3K4me3 profile at each stage and colors indicate ChIP-seq RPM.

In Figure S2, for male, Wk8, Wk15 and Wk23 hPGC samples are represented as early, middle and late stage; for female, Wk8, Wk14 and Wk21 hPGC samples are represented as early, middle and late stage, respectively. F, female; M, male.

**Figure S3, related to Figure 4, Chromatin state of DNA demethylation escapee genes in hPGCs**

(A) Genome browser view of H3K4me3 and H3K27me3 profiles on lncRNA *XIST*. Profiles were calculated as ChIP-seq RPM and smoothed as 3 pixels window using WashU Epigenome Browser. Data range was set as 1-fold to 10-fold of average RPM in each sample.

(B) Barplot showing *XIST* expression in hPGCs of both sexes at late stage.

(C) Pie plots showing the percentage of DNA demethylated and escapee 5kb-bin regions (top panel) and percentage of repeat-rich and repeat-poor escapee 5kb-bin regions (bottom panel) in hPGCs.

(D) Genome browser view of indicated histone modification and DNA methylation profiles at representative escapee gene *STX2* locus (shaded in light purple) and demethylated gene *DND1* locus (shaded in light yellow). Profiles are calculated as ChIP-seq RPM and smoothed as 3 pixels window using WashU Epigenome Browser. Data range is set as 1-fold to 10-fold of average RPM in each sample.

(E) Barplots showing *STX2* and *DND1* expression of both sexes at early and late stage.

(F) Boxplots showing the comparison of H3K4me3, H3K27me3, H3K9me3 ChIP-seq profiles, expression (FPKM) and DNA methylation level between escapee and demethylated promoters in male and female hPGCs at late stage.

In Figure S3, for male, Wk8-10 and Wk23 hPGC samples are represented as early and late stage; for female, Wk8-10 and Wk21 female hPGC samples are represented as early and late stage, respectively. F, female; M, male. Student's t-test was

performed to examine the significant statistical difference between two groups of data in boxplots. *$p < 0.05$, **$p < 0.01$, ***$p < 0.001$, n.s., not significant.

**Figure S4, related to Figure 4, Chromatin state of DNA demethylation escapee repeats in hPGCs**

(A) Barplot showing the DNA methylation (top panel) and expression (bottom panel) level rank of retro-transposable elements in hPGCs. Higher rank indicates higher DNA methylation and expression level. SVA, LINE1(L1) and hominoid-restricted ERVs (HERVs) are indicated in red.

(B) Boxplots showing the comparison of H3K4me3, H3K27me3, H3K9me3 ChIP-seq profiles, expression (FPKM) and DNA methylation level between escapee and demethylated repeats in male and female hPGCs at late stage.

(C) Scatter plots showing H3K4me3 and H3K9me3 profiles of SVA copies on human genome in male (left panel) and female (right panel) hPGCs at early and late stage. Red and blue dots represent SVA_A and SVA_E subfamily, respectively.

(D) Barplots showing histone marks, expression levels and DNA methylation levels in SVA subfamilies in male (top panel) and female (bottom panel) hPGCs at late stage. Left panel: barplot showing H3K4me3 and H3K9me3 preference of each copy of SVA subfamilies. Middle panel: barplot showing the expression level of each SVA subfamily. Right panel: barplot showing the DNA methylation level of each SVA subfamily.

(E) Barplots showing the normalized expression levels of LTR5_Hs/HERVK subfamily (left panel) and LTR14/HERVK subfamily in hPGCs.

(F) Boxplots showing the comparison of H3K4me3, H3K27me3, H3K9me3 ChIP-seq profiles, expression (FPKM) and DNA methylation level between proximal and distal genes relative to LTR5_Hs/HERVK subfamily (left panel) and LTR14/HERVK subfamily (right panel) in male (top panel) and female (bottom panel) hPGCs at late stage.

(G) Boxplots showing the expression levels between proximal and distal genes relative to LTR5_Hs/HERVK subfamily (left panel) and LTR14/HERVK subfamily (right

panel). Expression levels were represented as FPKM.

In Figure S4, for male, Wk8-10, Wk15 and Wk23 hPGC hPGC samples are represented as early, middle and late stage, respectively; for female, Wk8-10, Wk14 and Wk21 hPGC samples are represented as early, middle and late stage, respectively. Student's t-test was performed to examine the significant statistical difference between two groups of data in boxplots. *$p < 0.05$, **$p < 0.01$, ***$p < 0.001$, n.s., not significant. F, female; M, male.
